# Occupational stressors among firefighters: application of multi-criteria decision making (MCDM)Techniques

**DOI:** 10.1016/j.heliyon.2020.e03820

**Published:** 2020-04-23

**Authors:** Fazel Rajabi, Hossein Molaeifar, Mehdi Jahangiri, Shekofeh Taheri, Sean Banaee, Payam Farhadi

**Affiliations:** a Department of Occupational Health Engineering, School of Health, Shiraz University of Medical Sciences, Shiraz, Iran; b Research Center for Health Science, Institute of Health, Department of Occupational Health, Shiraz University of Medical Sciences, Iran; c College of Health Sciences, Old Dominion University, Norfolk, Virginia, USA; d Department of Management, Zand Higher Education Institute, Shiraz, Iran

**Keywords:** Psychology, Occupational stress, Firefighters, Delphi fuzzy method, Fuzzy analytical hierarchy process

## Abstract

**Background:**

Firefighters are exposed to a wide range of occupational stressors due to the nature of their job. Multi-criteria decision-making technique (MCDM) is a method for identifying, evaluating, and preventing occupational stressors among firefighters. The purpose of this study was to identify and prioritize the occupational stressors among firefighters using the fuzzy delphi method (FDM) and fuzzy analytical hierarchy process (FAHP).

**Methods:**

This qualitative-descriptive study was carried out in two stages. First, the most important occupational stressors of firefighters were identified and screened using a systematic review of scientific references and expert opinions based on the FDM. Then, all of the screened stressors were weighted and prioritized by the FAHP using the opinions of experts. All results were analyzed using Excel software.

**Results:**

Among, the 52 occupational stressors of firefighters identified in the first stage, 27 stressors were selected to enter into the FAHP. The FAHP results showed that among the four main dimensions, the weight of managerial factors (0.358) was more than other dimensions. The overall result of the study, regardless of the stressors’ main dimensions showed that the most important stressors among firefighters were financial strain due to inadequate pay, fear of explosion at incident scenes, toxic smoke, and gases produced at fires and lack of attention given to job safety by management, respectively.

**Conclusion:**

According to the results of the study, most occupational stressors of the firefighters are caused by organizational factors. Therefore, the implementation of management strategies to reduce the occupational stress of firefighters is recommended.

## 1 Introduction

Firefighting is a dangerous and stressful job due to direct exposure to harmful agents and stressful situations as well as high health risks (Burbeck et al., 2002; Ray et al., 2006). Studies have shown that in most cases, diseases and fatalities among firefighters are directly or indirectly related to the nature of their occupation. Research has also shown that occupational stress among firefighters is significantly higher than that in other occupations (Ziaei, Yarmohammadi, Izadi laybidi, Nazari and Hashemian, 2014). Studies show that firefighting ranks fifth in occupational mortality in the United States due to the dangerous and stressful situations that firefighters routinely encounter (Beaton and Murphy, 1993). Prevalence of occupational stress among firefighters is high since they directly deal with people's lives in very difficult conditions which require full awareness and on the spot decision-making (Kazronian et al., 2013).

Occupational stress has unpleasant impacts on employees and organizations including psychological, physical and organizational effects. Anxiety, depression, nervous exhaustion, irritability, aggression, sudden emotional unloading, overeating, impulsive behavior, inability to make decisions, poor concentration, distraction, and heightened sensitivity to criticism are some of the most important psychological effects of occupational stress (Darvishi et al., 2014; Magnavita, 2000). The most well-known physiological effects include migraine headaches, increased heart rate, hypertension, cardiovascular disease, musculoskeletal disorders, pulmonary disease, digestive disorders, kidney disease, rheumatoid arthritis, sleep disorders, headache and immune system disorders. Moreover, some important organizational effects of occupational stress include: absence from work, increased career turnover, low production, alienation of coworkers, job dissatisfaction, reduced commitment and loyalty to the organization, and decline in occupational performance and job quality. In addition, occupational stress may lead to inappropriate behavioral changes such as drug abuse and unsafe workplace behaviors (Darvishi et al., 2014; Hoogendoorn et al., 2002; LaDou and Harrison, 2007; Möller et al., 2005).

Considering the frequency of occupational stressors among firefighters and the serious outcomes and effects of these stressors, it is important to design and implement effective programs to reduce and control the stressors. However, the implementation of all stress management methods is not possible due to several financial and technical reasons. Identifying and prioritizing the stressors through multi-criteria decision making techniques (MCDM), which enables conversion of verbal concepts into mathematical terms (that facilitates decision-making and ranking of factors), is the best way to make informed and knowledge-based decisions and control occupational stress among firefighters. The MCDM techniques are based on mathematical and mental calculations and play an important role in overcoming uncertainties and making the right judgments based on the decision makers’ specific requirements (Kazemi et al., 2018; Sun, 2010). Today, the fuzzy analytical hierarchy process (FAHP) is one of the most widely-used decision-making methods. The FAHP is, in fact, the combination of the analytic hierarchy process (AHP) and fuzzy theory. The use of fuzzy theory enables users to make better judgments under conditions of uncertainty (Rokhsari and Sadeghi-Niaraki, 2015; Zadeh, 1965).

Considering the importance of firefighters' occupational stress, many studies have been conducted in various communities on this subject (Baghianimoghadam et al., 2015; Carpenter et al., 2015; Darvishi et al., 2014; ED, AA, & SM, 2013; Ha et al., 2008; Hoseinzadeh et al., 2013; M. G. Kim, Kim et al., 2013; Y.-K. Kim, Ahn, Kim, Yoon and Roh, 2016; Mehrabian et al., 2017; Monareh et al., 2018; Ocampos et al., 2017; Ray et al., 2006); however, few studies have focused on identifying and prioritizing occupational stressors among firefighters and there are no studies using multi-criteria decision-making methods. The present study aimed to identify and prioritize occupational stressors among firefighters using existing records and experts' opinions through FDM and FAHP techniques.

## 2 Method

This qualitative, descriptive and cross-sectional study, was conducted in two dependent phases. Firstly, Delphi Fuzzy Method (FDM) was used to identify and screen the most important stressors. Then, stressors determined in the first phase was prioritized and weighted using FAHP.

The study group consisted of two independent groups of experts (40 for DFM and 25 for FAHP). Expert panel members were selected purposefully from experienced full-time firefighters at least 10 years of work experience.

Notably, there are no explicit rules on how to select the number of experts in the MCDM, but the selection of panel members is done through non-probability sampling. In this method knowledge of the participant can be used to select the members of the group. The number of participants in most studies is less than 40 members qualified experts (Arof, 2015; Kil et al., 2016; Landeta, 2006; Powell, 2003; Rajabi et al., 2018).

The main stages of the study are as follows:

Phase 1 **Identifying and screening of occupational stressors**

**Figure 1:**
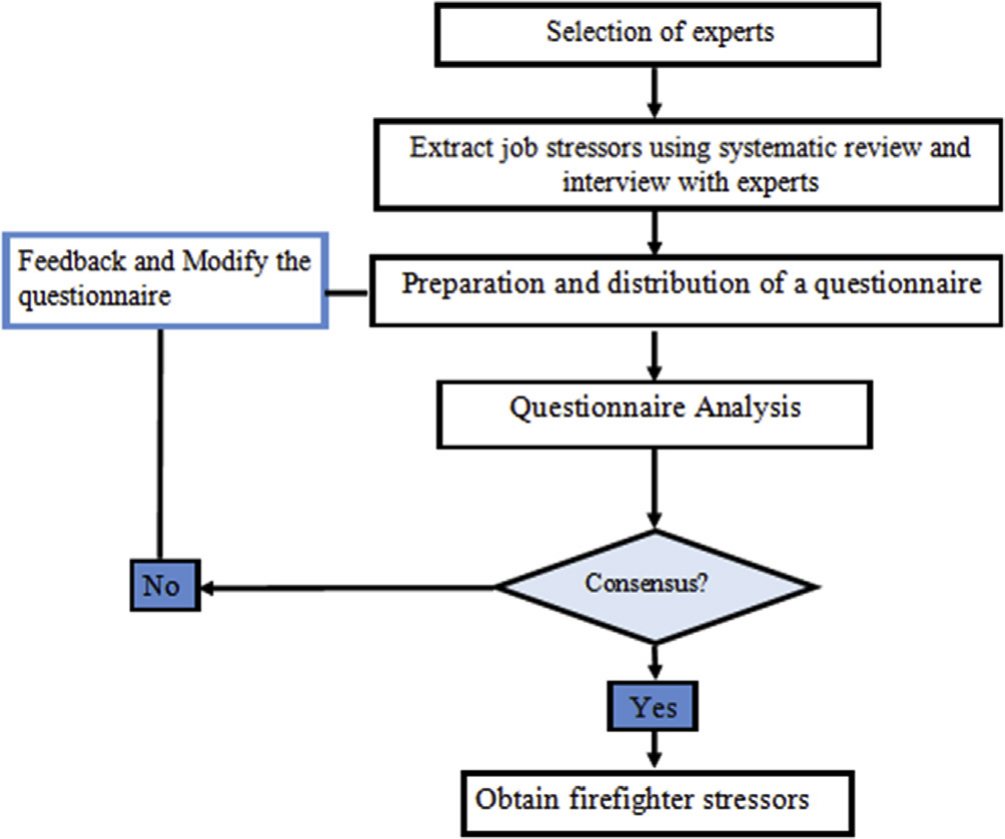
Steps of FDM in this study.

In the first step of this research, the most important occupational stressors of firefighters were extracted through the systematic review of scientific references (Baghianimoghadam et al., 2015; Beaton and Murphy, 1993; Carpenter et al., 2015; Ha et al., 2008; Hoseinzadeh et al., 2013; Kazronian et al., 2013; M. G. Kim et al., 2013; Y.-K. Kim et al., 2016; Mehrabian et al., 2017; Monareh et al., 2018; Saijo et al., 2007; Sawhney et al., 2017; Shantz, 2002; Stanley et al., 2018) and expert opinions based on FDM. Delphi technique is defined as a method for obtaining consensus using a series of questionnaires and providing feedback to participants (Dalkey and Helmer, 1963). This method is widely used where there is incomplete and uncertain knowledge about a topic. In fact, the basis of the Delphi method is the unbiased response to questions, the frequency of sending questionnaires and receiving feedback from them, and the final analysis of responses. The FDM was introduced by Kaufmann and Gupta in 1988. This method can overcome the ambiguities that exist in the opinions of the experts in the classical Delphi method (Kennedy, 2004; Roy and Garai, 2012). In this method, the linguistic scale is converted to fuzzy numbers, and for this purpose, triangular fuzzy numbers are used (Table 1). The steps of FDM in this study are shown in Figure 1.

**Table 1:** Linguistic variables and corresponding fuzzy numbers used in FDM (Phase 1) (Gumus et al., 2013).

Linguistic variables	Triangular fuzzy number
Very low important	(1,2,3)
Low important	(2,3,4)
Fairly low important	(4,5,6)
Medium important	(5,6,7)
Fairly high important	(7,8,9)
High important	(8,9,10)

**1. Selection of Experts:** In the Delphi studies, the group of experts should be selected from people who have sufficient knowledge and experience on the topic along with willingness and time to participate in the study (36). According to these criteria, 40 experts were selected for the first phase of the study.

**2.Extracting occupational stressors:** In the first step, the most important dimensions and sub-dimensions of firefighters' stressors were identified by semi-structured interviews with the expert group and systematic review of books, articles, and other credible scientific references. After the literature review and completion of initial expert interviews, similar and closely related suggestions were merged, and a new questionnaire was again given to experts. The new questionnaire was designed to obtain expert opinions based on fuzzy variables. In this study, the experts presented their opinions in a range of ‘very low’ important to ‘high’ important (Table 1).

**3 Analysis and review of questionnaires:** The questionnaire analysis was conducted according to the suggested options and linguistic variables. The mean experts' opinions were calculated using Eqs. (1) and (2):

(1) A^*i*^=(α1(i), α2 (i), α3(i), α4 (i)), i = 1,2,.....,n

(2) $$A_{m}=\left(\alpha_{m1}^{\left(i\right)},\alpha_{m2}^{\left(i\right)},\alpha_{m3}^{\left(i\right)},\alpha_{m4}^{\left(i\right)}\right)=\frac{1}{n}\sum \alpha_{1}^{\left(i\right)},\frac{1}{n}\sum \alpha_{2}^{\left(i\right)},\frac{1}{n}\sum \alpha_{3}^{\left(i\right)},\frac{1}{n}\sum \alpha_{4}^{\left(i\right)}$$

where, A^i^ is the opinion of i^th^ expert and A_m_represents the mean of expert opinions. Then the difference of each of the expert opinions from the mean was calculated using Eq. (3):

(3) $$\text{e}=\left(\text{α}{\text{m}}_{1}-{\text{α}}_{1}^{\left(i\right)}\text{,}\alpha {\text{m}}_{2}-{\text{α}}_{2}^{\left(i\right)}\text{,}\text{α}{\text{m}}_{3}-{\text{α}}_{3}^{\left(i\right)}\text{,}\alpha {\text{m}}_{4}-{\text{α}}_{4}^{\left(i\right)}\right)=\left(\frac{1}{n}\sum {\text{α}}_{1}^{\left(i\right)}-{\text{α}}_{1}^{\left(i\right)}\text{,}\frac{1}{n}\sum {\text{α}}_{2}^{\left(i\right)}-{\text{α}}_{2}^{\left(i\right)}\text{,}\frac{1}{n}\sum {\text{α}}_{3}^{\left(i\right)}-{\text{α}}_{3}^{\left(i\right)}\text{,}\frac{1}{n}\sum {\text{α}}_{4}^{\left(i\right)}-{\text{α}}_{4}^{\left(i\right)}\right)$$

After reviewing and modifying the initial questionnaire, another questionnaire was designed as indicated in the second round of FDM. In this questionnaire, the difference between the opinions of each of the expert were evaluated, compared, and the mean was presented. Then, questionnaires were distributed among the members of the expert group, they were asked to review their responses and, if necessary, to correct them. Then, the mean opinions of experts were calculated using previous equations.

In the final step, the values of each of the firefighter's occupational stressors were converted to non-fuzzy numbers using Eq. (4):

(4) $$S_{J}=\frac{u_{j}+m_{j}+l_{j}}{3}$$

4. **Determine the consensus between the experts**

In this study, Kendall's coefficient of concordance (W) was used to determine the degree of agreement among experts. The Kendall's W is a measure of the consensus between several judges (m) related to the N object or individual. This scale is particularly useful in evaluation of "validity among experts".The criterion for deciding whether to stop or continue the Delphi rounds is a strong consensus among panel members (Table 2) (Cafiso et al., 2013; Malekzadeh et al., 2015).

**Table 2:** Interpretation of Kendall's coefficient of concordance (Malekzadeh et al., 2015).

Kendall's coefficient value	0.1	0.3	0.5	0.7	0.9
Degree of consensus	Very weak	Weak	Moderate	Strong	Very strong
Confidence in the Priority of Factors	Not available	Low	Moderate	High	Very high

Phase 2 **Weighting and prioritizing occupational stressors using the FAHP method**

**Figure 2:**
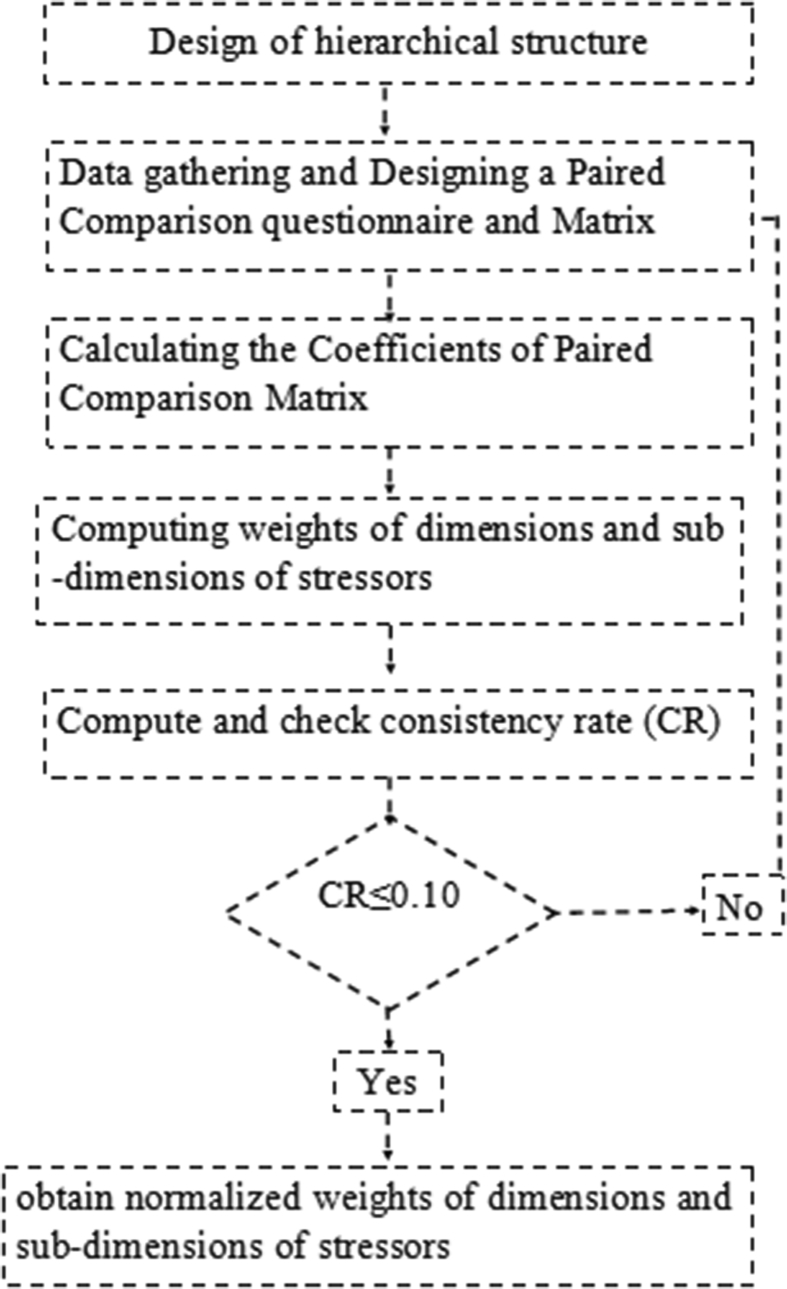
Steps of FAHP model.

**Figure 3:**
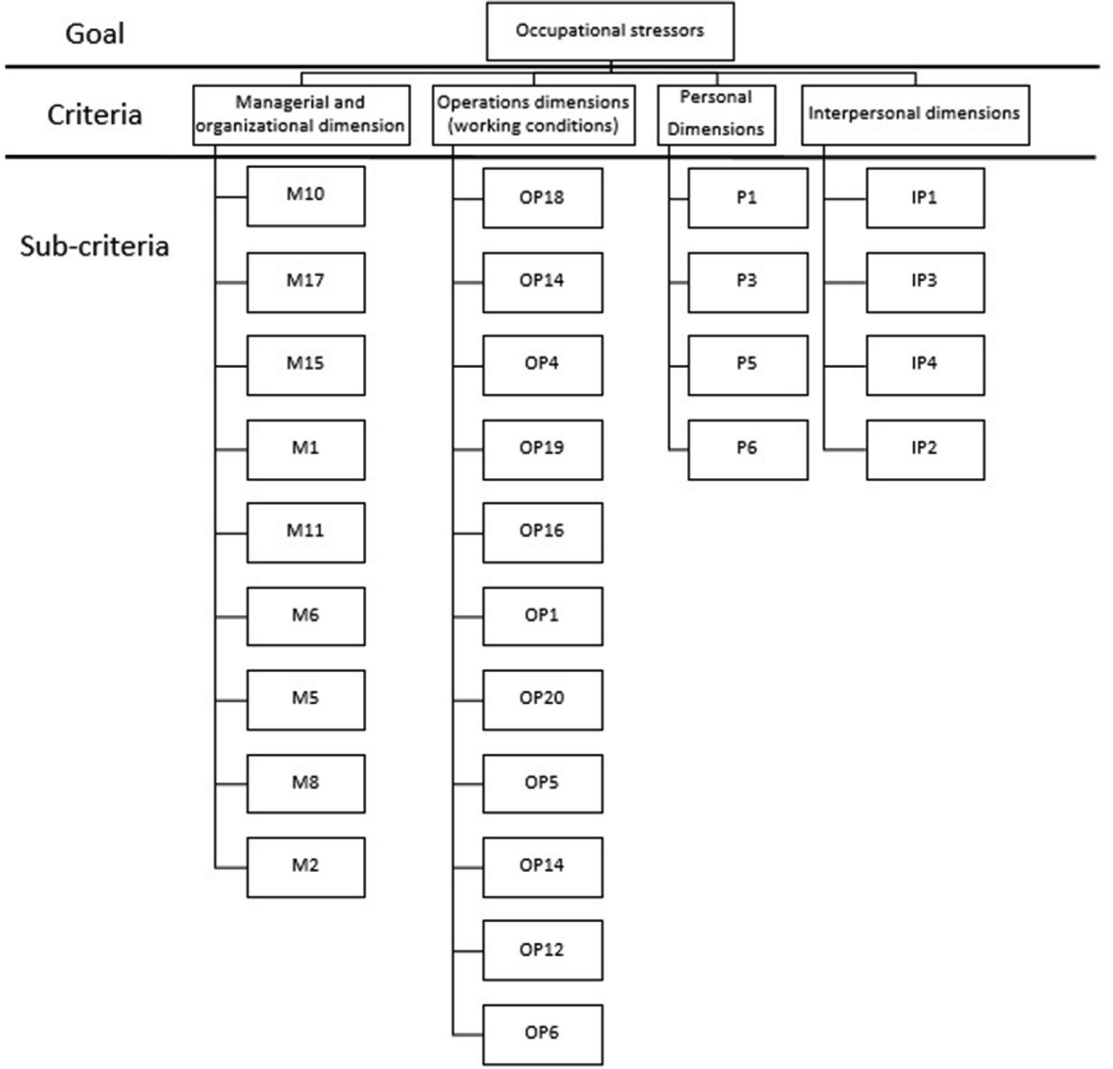
Hierarchical structure of the FAHP in this study.

In this phase, FAHP was used to prioritize stressors. FAHP is derived from the combination of the AHP method and fuzzy theory. AHP is the MCDM technique to analyze and organize complex decisions for evaluation and selection of alternatives within a set of criteria (Saaty, 1996). In AHP model, a problem is decomposed into a hierarchical structure where the goal is at the top, criteria are at the middle and alternatives are at the bottom of the hierarchy. However, AHP is ineffective when applied to determine inalienable vulnerability, uncertainty, and imprecision connected with the mapping of a decision maker's discernment with correct numbers (Chang, 1996; Tian et al., 2017). To overcome this limitation, FAHP has been utilized instead to address the inherent ambiguity in the assessment of the relative significance of characteristics and the performance ratings of alternatives with respect to characteristics (Chang, 1996; Sun, 2010; Van Laarhoven and Pedrycz, 1983). In other words, it is used to get crisp numerical values and rankings of subjective judgments. To perform FAHP calculations, the chang extent analysis (CEA) method was used. CEA is one of the simplest and most widely used methods in performing FAHP calculations (Chang, 1996; Kabir and Sumi, 2013; Mahdavi et al., 2015). This technique was also used by authors for classifying occupational stressors among nurses and farmers (Jahangiri et al., 2019; Rajabi et al., 2018). The steps of FAHP in this study were as follows (see Figure 2):

**A). Formation of a hierarchical structure:** To form a hierarchical structure, it is first necessary to determine its three main levels. The first and highest level in the hierarchical structure is to identify and prioritize the stressors in the firefighters. The second level of hierarchical structure includes defining indicators. In the present research, indicators were the same as the main dimensions of occupational stressors of firefighters. The third and lowest level of hierarchical structure, consists of the sub-dimensions of occupational stressors of the firefighters (see Figure 3).

**B). Designing a Paired Comparison questionnaire and Matrix:** At this stage, the stressor's dimensions and sub-dimensions that were screened by FDM, were formulated in a paired comparison questionnaire. In the next step, the questionnaires were completed by the expert panel. In this questionnaire, the importance of each stressor was determined in terms of linguistic scale. To perform fuzzy calculations, linguistic scales were converted to fuzzy triangular numbers (Table 3).

**Table 3:** Fuzzy linguistic scale and Triangular fuzzy number used in pairwise comparisons (Rajabi et al., 2018).

Linguistic Scale	Triangular fuzzy number	Reciprocal value of triangular fuzzy number
Exactly the same	(1,1,1)	(1,1,1)
The same	(1/2, 1, 3/2)	(2/3, 1, 2)
weak	(1,3/2,2)	(1/2,2/3,1)
strong	(3/2,2,5/2)	(2/5,1/2,2/3)
Very strong	(2,5/2,3)	(1/3,2/5,1/2)
definite	(5/2,3,7/2)	(2/7,1/3,2/5)

If triangular fuzzy numbers are represented in accordance with Eq. (5).

(5) Ai=(α_1(i)_, α_M(i)_, α_2(i)_), i = 1,2,......,n

where α_1(i)_ and α_2(i)_ are the minimum and maximum value, respectively; and α_M(i)_ is the most acceptable value.

The mean of the triangular fuzzy numbers derived from expert opinions is calculated according to Eq. (6):

(6) $$A_{ave}=\frac{A_{1}+\ldots A_{N}}{n}=\frac{\left(\frac{1}{n}\sum_{i=1}^{n}a_{1}^{\left(i\right)},\frac{1}{n}\sum_{i=1}^{n}a_{m}^{\left(i\right)},\frac{1}{n}\sum_{i=1}^{n}a_{2}^{\left(i\right)},\right)}{n}$$

Then, the pairwise matrix was designed using the fuzzy mean obtained in the previous step, as follows:

$$\tilde{A}=\left[\begin{array}{ccc} 1 & \tilde{{\text{C}}_{\text{ij}}} & \ldots \tilde{{\text{C}}_{\text{in}}}\\ \tilde{{\text{C}}_{\text{ji}}} & 1 & \ldots \tilde{{\text{C}}_{2\text{j}}}\\ \begin{array}{c} \ldots \\ \tilde{{\text{C}}_{1\text{i}}} \end{array} & \begin{array}{c} \ldots \\ \tilde{{\text{C}}_{2\text{j}}} \end{array} & \begin{array}{c} \ldots \ldots \\ \ldots 1 \end{array} \end{array}\right]$$

**C). Calculating the Coefficients of Paired Comparison Matrix**

After the data collection and formation of paired comparison matrices, the weights of the elements were calculated. To do this, the coefficients of each of the pairwise matrices were calculated using Eq. (7):

(7) $${\text{S}}_{\text{k}}=\;\sum_{\text{j}=1}^{\text{n}}{\text{C}}_{\text{kj}}^{\text{i}}\times \sum_{\text{i}=1}^{\text{c}}\sum_{\text{j}=1}^{\text{m}}{{\text{C}}_{\text{ij}}}^{-1}\;$$

where, k represents the number of the row, and i and j denote the alternatives and criteria, respectively. Then, the comparative magnitude of the fuzzy numbers was calculated. In general, if C_2_ and C_1_ are two triangular fuzzy numbers, their degree of relative importance is defined as (Eq. 8):

(8) $$\left[\begin{array}{c} V\left(M_{1}\geq M_{2}\right)=1\\ V(M_{1}\geq M_{2}=hgt\left(M_{1}\cap M_{2}\right) \end{array}\right]$$

**Figure 4:**
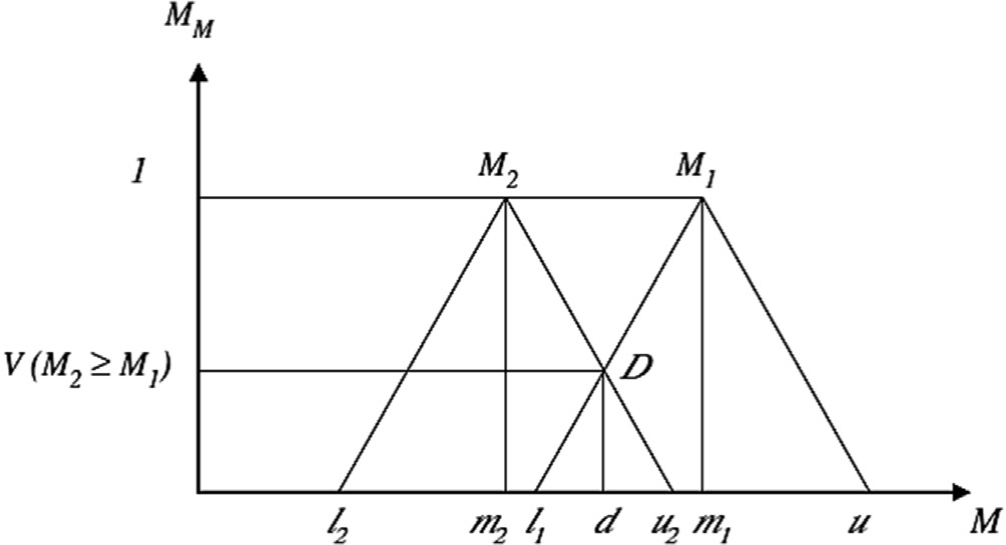
The comparison of two fuzzy number M1 and M2.

We also have Eq. (9) (see Figure 4):

(9) $$\text{hgt}\left({\text{C}}_{1}\cap {\text{C}}_{2}\right)=\frac{{\text{l}}_{1}-{\text{u}}_{2}}{\left({\text{m}}_{2}-{\text{u}}_{2}\right)-\left({\text{m}}_{1}-{\text{l}}_{1}\right)}$$

The degree of possibility (a triangular fuzzy number M to be greater than k triangular fuzzy numbers) was calculated from Eq. (10):

(10) $$\left({\text{M}}_{1}\geq {\text{M}}_{2},\ldots ,{\text{M}}_{\text{k}}\right)=\text{V}\left({\text{M}}_{1}\geq {\text{M}}_{2}\right),\ldots ,\text{ V}\left({\text{M}}_{1}\geq {\text{M}}_{\text{k}}\right)$$

D)**Compute weights of dimensions and sub-dimensions of stressors.**

The weight of dimensions and sub-dimensions of stressors in the paired matrix was calculated as follows (Eq.11):

(11) $${\text{W}}^{.}\left({\text{X}}_{\text{i}}\right)=\text{min}\left\{\text{V}\left({\text{S}}_{\text{i}}\geq {\text{S}}_{\text{k}}\right)\right\}\text{، K}=1\text{،}2\text{،}\ldots \text{nk}\neq 1$$

Therefore, the vector of dimensional weight is as follows (Eq. 12):

(12) $${\text{W}}^{.}={\left\{{\text{W}}^{.}\left({\text{X}}_{1}\right),{\text{ W}}^{.}\left({\text{X}}_{2}\right),{\text{ W}}^{.}\left({\text{X}}_{\text{n}}\right)\right\}}^{\text{t}}$$

Finally, the normalized weights of dimensions and sub-dimensions of firefighter's stressors was obtained from Eq. (13).

(13) $${\text{W}}_{\text{i}}=\frac{{\text{W}}_{\dot{\text{l}}}}{\sum {\text{W}}_{\dot{\text{l}}}}$$

The total weight of the stressors was also obtained by multiplying the weight of each stressor's sub-dimension by the corresponding dimension.

**E). Calculate Matrix Incompatibility Rate:**

The consistency of pairwise comparison matrix is one of the most important issues that should always be considered in the FAHP. In this study, the compatibility of judgments was evaluated by computing the consistency ratio as Eq. (14) (Ocampos et al.):

(14) $$\text{CR }=\;\frac{\text{CI}}{\text{RI}}\;$$

where, CI is the consistency index and RI is the average value of consistency index for random matrices (Table 4). The consistency index (CI) was computed using Eq. (15): (Bouzon et al., 2016; Mazurek, 2017)

(15) $$\text{CI}=\frac{Y_{max}-1}{n-1}$$

where, Y_max_ is the highest eigenvalue of the pairwise comparison matrix and n represents the size of the pairwise comparison matrix.

**Table 4:** Value of random consistency index (RI).

N	1	2	3	4	5	6	7	8	9	10
RI	0	0	0.52	0.9	1.12	1.24	1.34	1.41	1.45	1.49

The consistency ratio values less than 0.1 are considered acceptable (Ocampos et al.).

In this study, due to the large volume of computations and the necessity of their accuracy, all the above steps were done in Excel software.

## 3 Results

Table 5 presents the results of identifying and prioritizing occupational stressors among firefighters using Delphi method. The stressors with mean defuzzified values higher than the overall mean (7.4) were entered into the FAHP (see Table 6).

**Table 5:** Occupational stressors identified by firefighters in each dimension using the FDM and their average defuzzification values.

Main dimensions	Sub-dimensions	Code	Defuzzified mean opinion score
Interpersonaldimensions	Problematic relationships in the workplace	IP1	8.1∗
	Incorrect judgment from others about the performance of firefighters	IP2	8.1∗
	Being criticized by superiors and peers	IP3	8.2∗
	Lack of coordination between staff	IP4	7.7∗
	Neglect of colleagues to their job responsibilities	IP5	6.7
	Protect and care for people who do not cooperate	IP6	6.1
Operations dimensions (working conditions)	Traffic and low passageways	OP1	8.2∗
	Answer to radio in emergency situations	OP2	7.2
	Alarm noise, paging, and flashers	OP3	7.2
	Toxic smoke and gases produced in fires	OP4	8.2∗
	Heat produced from fire	OP5	7.4∗
	Exposure to contaminated and infectious agents	OP6	7.7∗
	Work in an unknown environment	OP7	5.5
	Work in adverse atmospheric conditions	OP8	6.7
	Work with substandard equipment	OP9	7.0
	Low speed and power of fire trucks	OP10	5.8
	Driving with high speed in an emergency condition	OP11	6.7
	Congestion in the incident scene	OP12	7.5∗
	Contact with contaminated objects	OP13	7.3∗
	Work in confined space	OP14	7.4∗
	Fear of falling objects	OP15	6.5
	Working at height	OP16	7.8∗
	Manual handling of heavy equipment	OP17	6.7
	Fear of explosion at incident scenes	OP18	7.9∗
	Watching a death and suffering from victims	OP19	8.4∗
	Arriving late to the incident scene	OP20	8.6∗
	Use of personal protective equipment (PPE)	OP21	6.8
	Failure in search and rescue operations	OP22	7.2
	Physical injuries during task	OP23	7.2
Personal Dimensions	Work-Life Conflict	P1	7.6∗
	Lack of interest in work at the fire department	P2	6.8
	Fear of making a mistake	P3	7.8∗
	Concerns about inadequate skills	P4	6.8
	Decision-making in emergency situations	P5	7.6∗
	Family and social issues affecting job performance	P6	7.8∗
Managerial and organizational dimension	Inappropriate schedule of rotational shift work	M1	7.6∗
	Poor management support	M2	7.8∗
	Not paying attention to the principles of ergonomics in the workplace	M3	6.8
	Role ambiguity	M4	6.8
	Role Conflict	M5	7.8∗
	High number of missions	M6	8.2∗
	Worries about job security	M7	6.6
	Lack of adequate place for rest	M8	7.8∗
	Shortage of equipment and resources for firefighting operations	M9	6.9
	Financial strain due to inadequate pay	M10	7.8∗
	Inequality between staff	M11	7.8∗
	Inadequate in-service training	M12	6.8
	Lack of opportunity for rest	M13	5.0
	Shortage of technicians for missions	M14	7.6∗
	Lack of appropriate nutrition to the firefighter job	M15	5.9
	Lack of attention given to job safety by management	M16	7.8∗

**Table 6:** The values of consistency Ratio for FAHP paired comparison matrices.

Matrix	CR
Interpersonal	0.024
Personal	0.052
Operations (working conditions)	0.012
Managerial and organizational	0.032
Total	0.0035

Kendall's coefficient of concordance (W) for the experts' responses to the order of factors in the second round of Delphi was 0.734, Which was significant at 95% confidence level. It reflects the strong consensus among experts.

**Figure 5:**
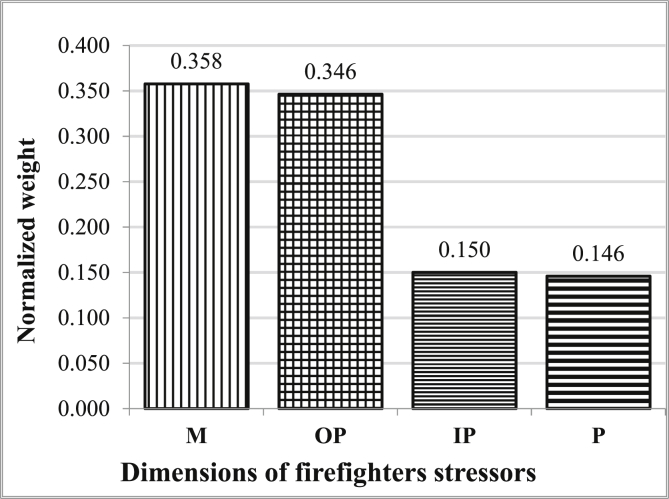
Prioritizing of main dimensions of the occupational stressors of the firefighters using the FAHP for stressors' code; refer to Table 5 (M: Management stressors, OP: Operation stressors, IP: Interpersonal stressor, P: Personal stressor).

Figure 5 shows the results of prioritizing the main dimensions of occupational stressors among firefighters using the FAHP method. As can be seen, managerial (0.385) and personal stressors (0.146) had the highest and the lowest weights among the four main dimensions of occupational stressors of firefighters, respectively.

**Figure 6:**
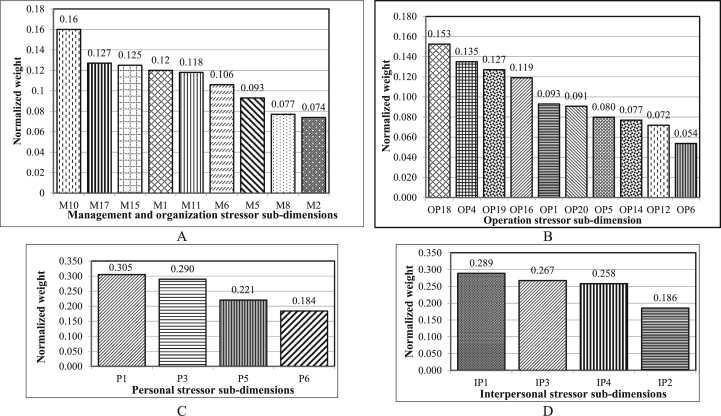
Prioritization of the occupational stressors among firefighters in each of the main dimensions using the FAHP method (A: Managerial Stressors, B: Operation Stressors, C: Personal Stressors, D: Interpersonal Stressors) (For stressors' code refer to Table 5).

**Figure 7:**
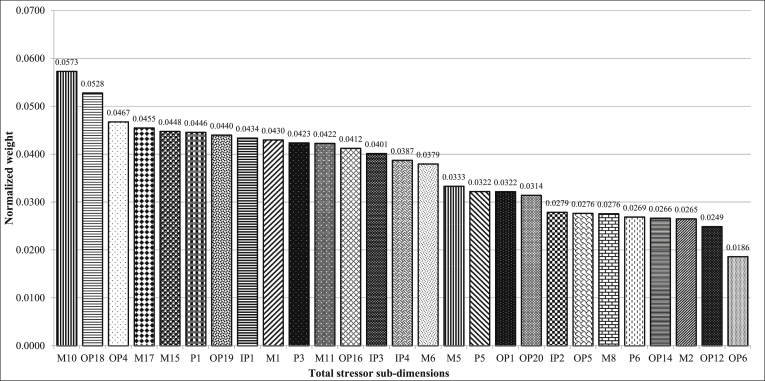
Overall Prioritization of stressors' sub-dimensions Regardless of the Main Dimensions of Stressors Using the FAHP (for stressors' code refer to Table 5).

Figure 6 displays the results of FAHP prioritization of sub-dimensions for various occupational stressors for each of the main dimensions among firefighters. As can be seen, fear of explosion at incident scenes, work-life conflict, problematic relationships in the workplace and financial strain due to inadequate pay had the highest weight among operation (workplace), personal, interpersonal and managerial dimensions, respectively. Prioritization of occupational stressors, regardless of their main dimensions, indicated that the most important stressors among firefighters were financial strain due to inadequate pay, fear of explosion at incident scenes, toxic smoke, and gases produced at fires, lack of attention given to job safety by management and shortage of technicians for missions, respectively (Figure 7). The values of CR for all pairwise comparison matrices were less than 0.1 (see Table 6). Therefore, the inconsistency of judgments was acceptable.

## 4 Discussion

In general, the purpose of this study was to investigate and prioritize occupational stressors among firefighters using FDM and FAHP. In this study, four dimensions of occupational stressors among firefighters (managerial, personal, interpersonal, and operational) were investigated. Based on the results of the research, managerial stressors had the highest weight among the main dimensions of occupational stressors in firefighters. “Financial strain due to inadequate pay" and "lack of attention given to job safety by management" were the highest priorities among managerial stressors in firefighters. "Fear of explosion at incident scenes" and " toxic smoke and gases produced at fires" had the highest weight among operation stressors. " Problematic relationships in the workplace” and "being criticized by superiors and peers" had the highest weights among interpersonal relation stressors. “Work-life conflict” and "fear of making a mistake" were the most important personal stressors. Prioritizing occupational stressors among firefighters, regardless of the dimensions, showed that “financial strain due to inadequate pay", "fear of explosion at incident scenes", "toxic smoke and gases produced at fires", "lack of attention given to job safety by management" and shortage of technicians for missions were the most significant occupational stressors among firefighters.

Although few studies have focused on the prioritization of occupational stressors among firefighters, they have worked through methods different from present study. Most of these results are consistent with the findings of the present study; however, some results are not consistent with our findings. This may be explained by differences among the stressors studied, among study methods and among statistical populations of the studies. The study of Darvishi et al. on firefighters in Sanandaj, Iran showed that the workplace's physical environment was the most important occupational stressor among firefighters (Darvishi et al., 2014). Kim et al. (M. G. Kim et al., 2013) introduced managerial and organizational stressors such as job insecurity, lack of reward, inappropriate occupational climate and job demand as the most important occupational stressors among firefighters in South Korea. Shantz (2002) identified the change in financial status, vacation, change in living conditions and personal injury or illness as the most important occupational stressors among firefighters. Monareh Yazdi (Monareh et al., 2018) found that occupational exposure to hazardous agents, concerns about inadequate skills, and the social support of co-workers and superiors were the most stressful occupational resources among firefighters. In another study by Kim et al. in South Korea, lack of rewards, inappropriate occupational climate, high job demand, job insecurity, interpersonal conflict, physical environment, and organizational injustice were introduced as the most important psychosocial stressors among firefighters (Ha et al., 2008).

In general, the aim of investigating and prioritizing occupational stressors among firefighters was to select optimal control measures for reduced occupational stress. As mentioned above, the managerial and organizational dimensions of occupational stressors had the greatest impact on firefighters. In addition, many other dimensions of occupational stressors presented in this study are also indirectly rooted in managerial and organizational stressors. Therefore, it is expected that management practices and organizational interventions can, to a large extent, reduce stress among firefighters. Based on the results of this study, the following measures are recommended to be taken:

• Increasing wages and rewards;
• Reducing employee workload by increasing the number of employees;
• Meeting health and safety requirements at the location of firefighting operations;
• Provision of fire-fighting facilities, in particular, safety devices and personal protective equipment;
• Describing job responsibilities to avoid ambiguity and conflict of roles;
• Modifying rotation of work shifts;
• Improving organizational climate through improved relationships, social support, sharing of views, and so on;
• Providing facilities at the staff resting place;
• Providing a good opportunity for employee involvement in decision making and reduced organizational hierarchy;
• Given that employee exposure to some stressors is unavoidable, individual interventions are required to aid in coping with stress. Meditation techniques, stress management training courses, support, and advice from a psychologist and passive attendance by a psychologist are some of the most important individual interventions for reducing occupational stress.

## 5 Limitations of the study and recommendations for future research

One of the important limitations of using multi-criteria decision-making methods is that there may be an error in their results due to the use of expert opinions. However, the use of two or more multi-criteria decision-making methods will increase the accuracy of the decisions made. Therefore, using other decision-making methods along with the FAHP method and comparing their results are recommended for future research. Also, given the diversity of the MCDM methods and the possibility of achieving different results, the use of sensitivity analysis for examining the congruence between the problem and the technique is recommended.

## Declarations

### Author contribution statement

F. Rajabi: Conceived and designed the experiments; analyzed and interpreted the data; contributed reagents, materials, analysis tools or data; wrote the paper.

H. Molaeifar: Conceived and designed the experiments; performed the experiments; contributed reagents, materials, analysis tools or data.

M. Jahangiri: Conceived and designed the experiments; analyzed and interpreted the data; contributed reagents, materials, analysis tools or data; wrote the paper.

S.H. Taheri: Conceived and designed the experiments; performed the experiments.

S. Banaee: Analyzed and interpreted the data; wrote the paper.

P. Farhadi: Analyzed and interpreted the data.

### Funding statement

This research did not receive any specific grant from funding agencies in the public, commercial, or not-for-profit sectors.

### Competing interest statement

The authors declare no conflict of interest.

### Additional information

No additional information is available for this paper.
